# Supplementary material for: Complexity of resting cortical activity predicts neurophysiological responses to theta-burst stimulation but fails to generalize: A rigorous machine-learning approach
Source: PLoS Comput Biol. 2026 Apr 30;22(4):e1014154. doi: 10.1371/journal.pcbi.1014154 (PMC13132237; doi:10.1371/journal.pcbi.1014154)
Supplement: S1 Text — (DOCX) [file pcbi.1014154.s001.docx]

# Complexity of Resting Cortical Activity Predicts Neurophysiological Responses to Theta-Burst Stimulation but Fails to Generalize: A Rigorous Machine-Learning Approach.

# Supplementary Methods & Materials

## Methods

### MRI Scans

Before the TMS sessions, T1-weighted anatomical MRI scans for neuronavigation during the TMS sessions were taken from participants. The full details of the experimental design for the Cohort 1 can be found in [1].

### Motor Hotspot

The following text is copied from [1]. “At the beginning of each session, the motor hotspot was determined over the hand region of the left motor cortex for eliciting motor-evoked potentials in the right FDI muscle. The hotspot was defined as the region where single-pulse TMS elicited consistent MEPs in the FDI muscle. Following International Federation of Clinical Neurophysiology (IFCN) guidelines, resting motor threshold (RMT) was determined on the FDI hotspot as the minimum stimulation intensity eliciting at least five MEPs (≥50 mV) out of ten pulses in the relaxed FDI using monophasic (posterior-anterior in the brain) current waveforms. In compliance with the IFCN safety recommendations, participants were asked to wear earplugs during hotspot and RMT trials to protect their hearing, and to minimize external noise. Active motor threshold was determined by again asking participants to flex their right index finger to engage the FDI muscle to approximately 20% of maximum voluntary contraction (MVC) and determined at the lowest intensity to produce MEPs of ≥200mv at least 5/10 times. TMS was administered with a thin layer of foam placed under the coil to minimize somatosensory contributions to TEPs. To minimize auditory evoked potentials related to the TMS coil click, auditory white noise masking was used throughout the TMS stimulation.”

### TBS Procedures

In both cohorts, each rTMS protocol was administered twice, one for the initial session and another for the retest session. For Cohort 1, five different rTMS protocols (1 Hz, 10 Hz, iTBS, cTBS and sham) were conducted for each participant whereas three rTMS protocols (iTBS, cTBS and sham) were conducted for Cohort 2. Thus, each participant in Cohort 1 underwent a total of 10 TMS sessions and similarly, 6 sessions for Cohort 2. The first block consisted of the initial sessions and the order was randomized for each participant. The second block consisted of retest sessions and the order from the first block was preserved in the second block. To minimize carry-over effect, the visits were spaced at least one week apart for Cohort 1 and at least two days for Cohort 2. Repeated sessions for each rTMS protocol were conducted at least 1 month apart in both Cohorts. Each participant underwent all sessions at roughly the same time of the day to control for possible circadian influences on the neuromodulatory effects of TMS. Only iTBS protocols are used for this report (Figure 1). All spTMS and rTMS protocols were applied to the left primary motor cortex (M1).

The following text is copied from [1]. “Participants were randomly assigned to receive either sham using either the cTBS or iTBS pattern, which was maintained across both sham visits. Sham cTBS and iTBS protocols were administered on the motor hot spot from the placebo side of the Cool-B65 A/P coil with a 3D printed 3 cm spacer additionally attached to the placebo side (MagVenture A/S, Farum, Denmark). Both active and sham-TBS protocols also included delivery of weak current pulses (between 2 and 4 mA and proportional to the intensity of actual TMS pulse) via surface electrodes (Ambu Neuroline 715 12/Pouch) placed approximately 1 cm below the inion bump and synchronized with the TBS trains to produce scalp sensations during both active and sham TBS conditions. This was done with the intention of blinding participants as to what kind of stimulation they were receiving when the direct somatosensory sensations of active TBS were not present during the sham stimulation.”

### rsEEG Preprocessing

In both data sets, rsEEG were first down-sampled to 250 Hz. In order, notch (band stop frequency 57-63 Hz, 4th total order forward-backward Butterworth) and high-pass (1 Hz high pass frequency, 4th total order forward-backward Butterworth) filters were applied. EEG channels contaminated by artifacts were manually identified and removed (Number of rejected channels reported as average ± SD. Cohort 1: 0.16 ± 0.44, Cohort 2: 0.64 ± 0.90. Number of remaining channels reported as average ± SD. Cohort 1: 58.84 ± 0.44, Cohort 2: 58.36 ± 0.90). A copy of continuous rsEEG at this step (after channel rejection but before epoch rejection) was saved for later. Then, continuous rsEEG was divided into 10-second epochs, visually inspected and bad epochs were manually recorded and removed. Number of rejected epochs are reported as average ± SD. Cohort 1: 1.70 ± 2.41, Cohort 2: 3.71 ± 2.61. Number of remaining epochs are reported as average ± SD. Cohort 1: 13.41 ± 2.60, Cohort 2: 12.77 ± 3.26. After that, EEG epochs were re-referenced to common average reference. Fast independent component analysis (fICA v2.5, http://research.ics.aalto.fi/ica/fastica/) EEGLAB plugin was used to compute independent components (ICs). Non-brain ICs that represented blink/eye movement, electromyographic activity, single electrode noise, or cardiac beats artifacts were manually identified based on their power spectrum, amplitude, scalp topography, and time course using TMS-EEG Signal Analyser (TESA v1.1.1, http://nigelrogasch.github.io/TESA) EEGLAB toolbox (Cohort 1: Total number of analyzed ICs – 59, average ±SD rejected ICs = 28.38 ± 5.92; total average ± SD remaining ICs = 29.73 ± 6.56. Cohort 2: Total number of analyzed ICs – 59, average ± SD rejected ICs = 28.71 ± 5.11; total average ± SD remaining ICs = 28.67 ± 4.96). The ICA weights obtained were then applied to continuous rsEEG saved before. Channels rejected during the previous steps were interpolated using spherical interpolation. Next, continuous rsEEG was band-pass filtered with [1, 40] Hz as the passband edge frequencies (4^th^ total order forward-backward Butterworth). Finally, the continuous rsEEG was divided into 10-second epochs and epochs rejected in previous step were also rejected in this step.

### MEP Preprocessing

For both studies, monopolar EMG data from the belly and tendon of the right hand’s first dorsal interosseous (FDI) muscle was acquired with surface electrode pairs (made of Ag-AgCl) with the ground electrode located on the right ulnar styloid. These EMG data was digitized at a sampling rate of 5 kHz by the amplifier system (BrainVision actiCHamp system, Brain Products GmbH) and BrainVision Recorder software (Software Version 1.20.0601, Brain Products GmbH). Epochs were extracted with a 150ms window extending 50ms before and 100ms after the spTMS.

EMG data was preprocessed offline using custom MATLAB script (MATLAB R2022b, Math-Works Inc., USA). EMG data was first band-pass filtered (10-2000 Hz). Then, for each epoch, baseline correction was applied (by subtracting the mean of -50 to -5 ms window relative to spTMS from the entire EMG epoch). Next, root mean square (RMS) of EMG signal was computed using -20 to 13 ms window relative to spTMS (excluding -2 to 2 ms period to exclude any TMS artifacts). Epochs with RMS 2.5 standard deviation above the mean RMS of the entire block were excluded from further analysis. The peak-to-peak amplitude of MEP was computed for the remaining epochs using the 18 to 50 ms window.

### TMS-EEG Preprocessing

Users can refer to [1] for the full details of TMS-EEG preprocessing. TMS-EEG data was preprocessed offline using custom MATLAB script (MATLAB R2017b and MATLAB R2021a, Math-Works Inc., USA). All trials were segmented into 3000 ms epochs, extending 1000 ms before and 2000 ms after spTMS. Baseline correction was applied as followed: the mean of the -900 to -100 ms window (relative to spTMS) was subtracted from the entire epoch in each channel. Next, epochs were visually inspected and noisy channels identified and removed (number of rejected channels reported as average ± SD. Cohort 1: 2.5 ± 1.5, range 0-4 out of 63, Cohort 2: 3.9 ± 3.3, range 0-14 out of 63). Zero-padding was applied to the -2 to 14 ms window to remove early TMS pulse artifact. Thereafter, voltage statistics including amplitude (≥100 μV), kurtosis (≥3) and joint probability (single-channel-based threshold ≥ 3.5 SD; all-channel-based threshold ≥ 5 SD) were computed to automatically identify noisy epochs before a manual review for removing noisy epochs. (Number of rejected epochs are reported as average ± SD. Cohort 1: 18 ± 6, range 2-39 out of 120, Cohort 2: 22.7 ± 16.8, range 0-75 out of 150). An initial round of fast independent component analysis (fICA) was performed for blind-source segregation of TMS-EEG into components. Components were visually reviewed and identified for early TMS evoked high amplitude electrode and EMG artifacts (Number of components rejected are reported as average ± SD. Cohort 1: 1 ± 1, range 0-3 out of 63, Cohort 2: 1 ± 0.4, range 0-2 out of 63). The remaining components were mapped back to sensor space, its zero-padded time window linearly interpolated (-2 to 14 ms). In order, the band pass filter (forward-backward 4^th^ total order Butterworth filter from 1 to 100 Hz) and notch filter (forward-backward 4^th^ total order Butterworth filter between 57 and 63 Hz) were applied. TMS-EEG were referenced to common-average reference. 2^nd^ round of fICA was run to manually identify and remove noisy components that include eye movements and blinking, muscle artifact (EMG), single electrode noise, TMS evoked muscle and cardiac beats (EKG). (Number of components rejected are reported as average ± SD. Cohort 1: 1 ± 1, range 0-3 out of 63, Cohort 2: 25.7 ± 4.9, range 13-3 out of 63). For Cohort 1, auditory evoked potential was removed whereas for Cohort 2, auditory evoked potential was left intact. Spherical interpolation was applied to the remaining missing channels before low pass filter (4^th^ total order forward-backward Butterworth filter at 50 Hz) was applied. Trials were further segmented to 500 ms before and 1000 ms after spTMS.

### Data Normalization

All features were normalized either using the z-score using the mean and standard deviation of the training set or the distances to the median defined as follows:

$\tilde{x}=\left| \log_{10} x-\log_{10} median(\boldsymbol{X}) \right|$,

where **X** represents the training set and will be called Log-Distance transformation.

### Feature Grouping and Model Selection:

Here, we adopt manual feature grouping for feature and model selection. Feature groups are iteratively generated through different combinations of regions of interested (ROIs), data transformations and either powers of frequency bands, measures of complexity or both (Figure 2). Thus, examples of feature groups include 1) standard normalization of the alpha band powers of rsEEG from the central region, 2) distances to the medians transformation applied to coarse-graining multiscale sample entropy of rsEEG from the left motor region, and 3) distances to the medians transformation applied to both beta band powers and approximate entropy of rsEEG from the whole-scalp region. Each feature group represents a set of features to be trained by a classifier. Next, models are iteratively generated by generating all different combinations of feature groups, classifiers and categorization methods of the modulation of corticospinal or cortical excitability.

Thus for the MEP Experiment, a total of 10,152 models (9 types of classifier × 3 ROIs × 2 transformations × 2 post-iTBS MEP blocks (T5 vs T25 blocks) × (4 frequency bands + 18 measures of complexity + (4 × 18)) were trained in cross-validation for the model selection step whereas for LMFP Ratios Experiment, a total of 218,268 models were trained in cross-validation for the model selection step (9 types of classifier × 3 ROIs × 2 transformations × 43 LMFP windows × (4 frequency bands + 18 measures of complexity + (4 × 18)).

### Chance-Level Performance

The formulas for chance-level metrics for imbalanced 2-class sample are as followed:

Accuracy = prob_pos * samp_pos + prob_neg * samp_neg

Sensitivity = (prob_pos * samp_pos) / (prob_pos * samp_pos + prob_neg * samp_pos)

Specificity = (prob_neg * samp_neg) / (prob_pos * samp_neg + prob_neg * samp_neg)

AUC ROC = 0.5

F1 score = (2 * prob_pos * samp_pos) / (prob_pos + samp_pos)

AUC PR = samp_pos

PPV = (prob_pos * samp_pos) / (prob_pos * samp_pos + prob_pos * samp_neg)

NPV = (prob_neg * samp_neg) / (prob_neg * samp_pos + prob_neg * samp_neg)

where prob_pos is the probability of the label being positive, prob_neg is the probability of the label being negative, samp_pos is the proportion of the sample being positive, samp_neg is the proportion of the sample being negative.

### Feature Importance

In LMFP Ratios Experiment, for the decision tree, the feature importance for each feature is estimated using the Gini importance, which is defined as the total reduction of the Gini impurity brought by that feature. This is only available for the decision tree.

## Results

### MEP and LMFP Cross-Session Experiments

Briefly, for the MEP Cross-Session Experiment, LDA CV trained on the complexity index of composite multiscale distribution entropy (using Log-Distance transformation) from EEG channels in the left motor region and pre-TBS features, using T25 t-test as the categorization method, has the highest ROC-AUC (mean ± 95% confidence interval: 75.0 ± 6.9, accuracy: 71.3 ± 7.4, sensitivity: 66.0 ± 10.8, specificity: 82.0 ± 11.4, precision-recall AUC: 82.3 ± 4.6, Supplementary Table S4, row a). When tested on the test set, the performance substantially fell in all metrics (ROC-AUC: 53.6 [36.7, 74.5], accuracy: 52.8 [36.1, 69.4], sensitivity: 47.4 [28.8, 65.9], specificity: 58.8 [35.3, 76.5], precision-recall AUC: 62.7 [51.6, 80.1]) (Supplementary Table S4, row b).

For the LMFP Cross-Session Experiment, when trained on the complexity index of coarse-graining multiscale distribution entropy (using Log-Distance transformation) from the left motor region and pre-TBS features to the TEP ratios using the 105-135 ms window, Logistic Regression with Lasso regularization has the highest cross-validated ROC-AUC (mean ± 95% confidence interval: 83.8 ± 7.4, accuracy: 89.7 ± 4.8, sensitivity: 97.6 ± 2.6, specificity: 70.0 ± 14.1, precision-recall AUC: 90.0 ± 4.4, Supplementary Table S4, row c). Again, when tested on test set, the performance dropped substantially in all metrics (ROC-AUC: 48.6 [39.2, 65.1], accuracy: 60.5 [49.9, 71.1], sensitivity: 90.0 [75.0, 100.0], specificity: 27.8 [16.7, 44.4], precision-recall AUC: 50.8 [48.2, 69.1], Supplementary Table S4, row d), with skew to high sensitivity and low specificity.

The performance drops suggested overfitting but both models from Cross-Session Experiments were relatively small (10 features for MEP Experiment and 8 features for LMFP Experiment) and had regularization. As post-hoc analysis, we performed statistical and reliability analysis, presented in the next two sections.

Cross-Subject LMFP Experiment

The model with the highest cross-validated ROC-AUC is the decision tree trained on the complexity indices of time-shifted multiscale distribution entropy (using Log-Distance transformation) from the central region, with the participants classified using the LMFP ratios computed using the 85-115 ms window (mean ± 95% confidence interval: ROC-AUC: 75.0 ± 5.1, accuracy: 75.3 ± 5.2, sensitivity: 78.7 ± 8.4, specificity: 71.4 ± 7.8, precision-recall AUC: 74.2 ± 5.1) (Supplementary Figure S3). When tested on the external validation cohort, the performance dropped to chance-levels (ROC-AUC: 50.6 [41.0, 65.3], accuracy: 50.0 [40.3, 63.6], sensitivity: 54.5 [45.5, 77.5], specificity: 46.7 [33.3, 67.0], precision-recall AUC: 42.6 [38.7, 52.1]) (Supplementary Figure S4).

1. Ozdemir RA, Boucher P, Fried PJ, Momi D, Jannati A, Pascual-Leone A, et al. Reproducibility of cortical response modulation induced by intermittent and continuous theta-burst stimulation of the human motor cortex. Brain Stimul. 2021;14:949–64.
