# Supplementary material for: Complexity of resting cortical activity predicts neurophysiological responses to theta-burst stimulation but fails to generalize: A rigorous machine-learning approach
Source: PLoS Comput Biol. 2026 Apr 30;22(4):e1014154. doi: 10.1371/journal.pcbi.1014154 (PMC13132237; doi:10.1371/journal.pcbi.1014154)
Supplement: Attachment — Submitted filename: Review PLoS CompBio Nov25.docx [file pcbi.1014154.s012.docx]

**Review for PLoS Computational Biology on „Complexity of Resting Cortical Activity Predicts Neurophysiological Responses to Theta-Burst Stimulation but Fails to Generalize: A Rigorous Machine-Learning Approach.“**

15. November 2025

Reviewers:

David Menrath, M.Sc.	ORCID: 0009-0001-9291-8550

Joshua P. Woller, M.Sc.	ORCID: 0000-0002-5164-4311

**Introduction:**

The presented study aims to address the limited clinical efficacy of intermittent theta-burst stimulation (iTBS) treatments, which by now still exhibits strong response variability, by focusing on neural biomarkers to predict treatment responsiveness. Doing so, the authors correctly identify a number of important issues of current neurostimulation treatments, namely inter individual response variability, non-validated biomarkers for response prediction, lack of mechanistic understanding of TMS treatments and especially the lack of studies investigating intra individual rest-retest reliability of treatment responses. To address this, the authors present two datasets of intra-subject repeated measures, highly suited to address the issues. The datasets are well suited to add to the mechanistic understanding of iTBS TMS treatments.

While we strongly agree with the author’s view on the importance of the aforementioned issues, and the value suitability of their datasets to answer these questions, we identify substantial problems with the conceptualization and implementation of their analysis. This review thus focusses on these conceptual and statistical choices on a macroscopic level. Furthermore, we see some problems with later analysis choices but see them as secondary and will address them in the later part of the review.

For this review we have split our concerns into major and minor concerns. We consider the major concerns as necessary to address before publication of the manuscript. We believe that addressing the minor concern would further increase the scientific quality of the manuscript.

**Major Remarks**

***Conceptualization and Statistical Choices***

**CS1**

We consider the problem to be correctly identified, but the statistical treatment of it in the manuscript to be in error. We do not share the authors view of the presented problem (variability of iTBS efficacy) as a classification problem and would advise against predicting treatment success as a dichotomous variable. It is highly unlikely that effects of iTBS treatments are bimodally distributed, showing stereotyped, strong effects in one subgroup and no effects at all in another. We would ask the authors to provide sources and the neurological rationale for this framing as a binary classification task.

This is made even more problematic by the fact that data are not intrinsically labeled. The dichotomization is derived from an ad-hoc statistical procedure, namely, subjects are categorized based on the statistical significance of a t-test between the observed MEPs. Regarding the analysis of MEPs using peak-to-peak measures, it has to be noted that peak-to-peak measures are statistically non-robust and prone to the influence of noise and outliers.

We believe that analyzing TMS efficacy in their datasets as a regression problem could lead to better mechanistic insights as we find it reasonable to model graded responses to brain stimulation; if it is true that some people do not respond to treatment at all, zero-inflated models could prove to be a useful consideration.

**CS2**

We believe that the study might suffer from an inflated number of false negative non-responders as the authors use a two-sample t-test (instead of a paired t-test) for a repeated measures design. The too large variance estimate of the two-sample t-test will produce inflated p-values that might falsely label responders as non-responders. This is especially detrimental in connection with the classification problem from **CS1** as responders and (falsely labeled) non-responders might share features that could predict treatment success but then would falsely lose predictive power.

Further, there is no specific reason why a p-value threshold of 0.05 should distinguish responders from non-responders. In fact, this threshold constitutes an additional, hidden parameter with possibly high leverage on the results and their interpretation, and is mere convention in frequentist statistics about the acceptable false-positive rate in the absence of any true effect. Even when considering the p-values as suitable dichotomization variable, the distribution of the t-statistics would be necessary to validate the assumption of a bimodal distribution.

**CS3**

In general dichotomization without a safety margin is problematic as it leads to a loss in discriminative power, especially when looking at graded effects. Without a safety margin around the decision threshold, insignificantly different subjects are classified as responders and non-responders, making it difficult to extract valuable predictors, as they are close in the feature space, muddying the waters effectively.

We would therefore like to see the distributions of MEP amplitudes and LMFP ratios that were used for either responder and non-responder population labelling or the labeling of facilitation or suppression.

***Signal Processing***

**SP1**

When employing machine learning in neuroscience, interpretability of features matters at least as much as predictive accuracy. The authors claim that the study shows the superiority of complexity measures over regular time or frequency domain analyses. While complexity measures are an interesting upcoming analytical tool, we do not fully see that claim justified based on their data because the complexity measures are the only analysis features that make use of the full time series nature of the data.

We think the measures employed from time or frequency domain are insufficient. They, in contrast to the complexity measures, rely on heavy averaging and do not make sufficient use of the time series structure or trial structure of the data. Including both time series analyses (making use of the prototypical response beyond max amplitude point measures) and network features such as dynamic connectivity, phase amplitude couplings to characterize network state into the models might further increase interpretative, discriminative or predictive power for iTBS efficacy.

It remains vague how complexity measures based on scalp EEG in clinical applications of neurostimulation actually reflect endogenous brain activity, compared to how much is contributed by muscle or stimulation artifacts. Given these interpretative problems, the predictive power of complexity measures cannot support the scientific advancement of EEG-based TMS interventions.

**Minor Remarks**

***Signal Processing***

Neighboring EEG channels share a lot of signal but usually have a unique noise component. Hence to use the spatially distributed source activity, spatial filtering can be utilized to maximize signal to noise ratios. We would generally advise against single channel-based features and pure averaging of channel clusters.

**Closing statement**

We believe the paper and especially the two collected datasets possess high potential to advance iTBS TMS research. We believe that implementation of the aforementioned major remarks might change the outcomes and interpretations compared to the current state of the manuscript. While we would have further remarks to the authors choices in details of signal processing, plotting and statistics, we consider it necessary to address those only if the major remarks have been implemented. We look forward to the revised manuscript.
