# Supplementary material for: Complexity of resting cortical activity predicts neurophysiological responses to theta-burst stimulation but fails to generalize: A rigorous machine-learning approach
Source: PLoS Comput Biol. 2026 Apr 30;22(4):e1014154. doi: 10.1371/journal.pcbi.1014154 (PMC13132237; doi:10.1371/journal.pcbi.1014154)
Supplement: Attachment — Submitted filename: PLOS_CompBiology_Response2Reviewers_v04.docx [file pcbi.1014154.s014.docx]

Response to Reviewers

We would like to thank the editors and all of the reviewers for spending the time to review our manuscript and provide constructive comments. We also thank the editors for giving us the opportunity to revise our manuscript. We understand that our original manuscript can be confusing and hard to follow so your effort to review it is deeply appreciated. We’ve updated our entire manuscript in accordance with the suggestions provided, which has greatly strengthened our manuscript. Below is the summary of the changes, as well as individual comments from all of the reviewers:

## Summary:

We’ve made significant revision to the manuscripts. Below is a summary of major revisions:

- We’ve revised our conclusion that concept drift only partially explain the poor performance of our machine learning model in cross-session experiment.
- We’ve significantly revised Figure 2, 3 and 4 to improve clarity.
- We’ve explicitly stated the problem to be a binary classification in both the abstract and introduction section.
- We’ve replaced some lesser-used acronyms with their full spelling.
- We’ve added formulas in the Method Section.
- We’ve added 4 more figures to the Supplementary Methods and Materials.

## Reviewer 1’s Summary:

In this work, authors use ML methods to predict neurophysiological responses to iTBS session. The result show promising prediction performance, however, the overall quality and impact can be significantly improved by addressing the following concerns.

## Reviewer 1’s Comments:

**C1: Authors highlighted that most machine-learning (ML) studies have focused on modeling behavioral or clinical effects of repetitive transcranial magnetic stimulation (rTMS), the few studies examining neurophysiological outcomes, so the proposed work fills this gap. However, it is important to discuss more about why it would be important to do the neurophysiological outcomes prediction and its responding challenges. Missing those discussion is misleading since focusing on behavioral or clinical effects of rTMS helps to address the challenges in application, which is essential for many fields.**

R1: We thank the reviewer for highlighting this critical gap. We agree that justifying the focus on neurophysiological outcomes is essential, especially given the ultimate goal of clinical application. As the reviewer notes, rTMS and particularly Theta Burst Stimulation (TBS) are now in daily use for treating various psychiatric and neurological populations. The clinical efficacy of these protocols was largely attributed to the "Cortical Excitability Hypothesis", the assumption that iTBS consistently facilitates and cTBS suppresses cortical activity via Hebbian-like plasticity mechanisms (LTP/LTD), and that modulation of excitability drives the subsequent behavioral improvement.

However, accumulating evidence reveals high inter-individual variability in these neurophysiological responses. If the fundamental assumption (i.e., that iTBS facilitates excitability) does not hold for a specific patient/subject due to their initial brain state, the downstream clinical effects may become unpredictable/inconsistent as well. Therefore, focusing solely on behavioral/clinical prediction treats the brain as a "black box" and ignores the mechanistic link. By targeting neurophysiological outcomes, we aim to bridge this gap. Predicting these outcomes allows us to (1) test whether baseline electrophysiological state explains variability in iTBS-induced physiological change (2) help identify who is likely to show target engagement before engaging in lengthy clinical courses, and (3) ultimately provide a principled route to individualizing stimulation parameters to improve efficacy. We have significantly expanded the Introduction and Discussion sections:

Specifically, an expanded motivation background was included in the introduction section as follows;

“While predicting clinical response is essential, it often bypass the physiological mechanisms. Given that clinical efficacy is assumed to depends on whether the intended neurophysiological mechanism was engaged, focusing solely on clinical outcomes without characterizing the intermediate physiological responses leaves a critical gap in our understanding. If the expected neurophysiological modulation fails to occur due to the individual’s baseline state, the subsequent clinical benefit may be compromised. As such, neurophysiological outcomes such as MEPs and TMS-evoked EEG potentials (TEPs) provide more proximal indices of physiological “target engagement” and offer a mechanistic bridge between baseline brain state, stimulation, and downstream behavior. Predicting these outcomes allows us to test whether baseline electrophysiological state explains variability in iTBS-induced physiological change, identify individuals likely to show target engagement before lengthy treatment courses, and motivate principled individualization of stimulation parameters to improve efficacy.” (page 7, line 17-23, page 8, line 1-6 of the revised manuscript).

We also emphasized our motivation at the beginning of the discussion as follows.

“Given the widespread clinical use of TBS and the long-standing assumption that patterned stimulation produces reliable physiological target engagement, we investigated whether baseline electrophysiology can predict the direction of iTBS-induced neurophysiological change and whether such prediction generalizes across independent samples” (page 27, line 1-4 of the revised manuscript).

**C2: Although the model is doing neurophysiological outcomes prediction, but based on the methods, it is still a classification model. The manuscript should reflect this clearly and mention this as one of its potential limitations. In most ML studies about response prediction, the model should intend to predict the signal response as a continuous variable.**

R2: Based on your suggestion, we’ve clarified in both the Abstract (page 2, line 15-16) and Background sections in page 9 line 6-9 of the revised manuscript. Please also see our responses to Reviewer 2 for discussion on treating it as a regression vs classification problem.

**C3: The details of the Schematic Diagram (Fig 2) need to be improved. It's unclear what would be the input and what would be the output of the model (especially the output, and how the output is defined).**

R3: Thank you for providing us helpful tips on improving our Schematic diagram. Based on your suggestions, we’ve significantly revised Figure 2 for clarity. Specifically, we’ve labelled features as the input (x) and categorization method as the labels (y).

**C4: In the method section, authors should include the sample size (the number of trials) used in the model and have a detailed introduction.**

R4: We’ve already reported the sample size in the Method section, please refer to page 17, line 4-10 of the original manuscript.

**C5: For the cross-fold validation, did the author use leave-one-subject out methods? The current compelling result might be a result of data leak, since it might just simply learning the subject information (i.e., predict based on recognizing the subject).**

R5: We appreciate this rigorous question. To clarify, in Experiment 1 (Cross-Session), the training set consisted exclusively of initial visits (one session per subject), making subject leakage impossible during internal cross-validation. Also initial and retest sessions are conducted at least one week apart to minimize carryover effect. In the Cross-Cohort Experiment, the external validation set (also known as the test set) contains subjects unseen by the model during training. Given the low test-retest reliability of outcome measures across sessions, potential session data leakage from a given subject in Cross-Cohort Experiment would possibly compromise internal validation performance.

**C6: Is the label (y) of the model being balanced?**

R6: While most of the response variables are balanced, a few are not. In all cases, we used stratified cross-validation. We also computed theoretical chance-level for each metrics based on the class proportion, but added their formulas in Chance-Level Performance Section on page 4-5 of the revised Supplementary Methods and Materials.

**C7: Authors should include a baseline model as a control and compare its result with the included ML method. If an fair chance (such as 50% for binary or 33% for 3-class) can be reported, then address point 6 and authors can report the pair chance as control (but even after addressing point 6, a baseline model is still preferred because of the fuzzy nature of neurophysiological outcomes).**

R7: We already included the theoretical chance-level for a given class proportion, reported as dark horizontal lines in Figure 4. We apologized for not including the details before but we added the details for computing theoretical chance-level performance, which we took to be the baseline performance, in Chance-Level Performance Section on page 4-5 of the revised Supplementary Methods and Materials.

**C8: The provided GitHub link is expired and the dataset link is not directing to the dataset itself but the hosting platform.**

R8: We apologized for the technical errors. The GitHub and dataset links are now accessible to public.

## Reviewer 2’s Summary:

The review is uploaded as an attachment. We like the conceptual and empirical groundwork but think certain statistical choices are necessary to make it more interpretable and robust.

## Reviewer 2’s Comments:

**C1: We consider the problem to be correctly identified, but the statistical treatment of it in the manuscript to be in error. We do not share the authors view of the presented problem (variability of iTBS efficacy) as a classification problem and would advise against predicting treatment success as a dichotomous variable. It is highly unlikely that effects of iTBS treatments are bimodally distributed, showing stereotyped, strong effects in one subgroup and no effects at all in another. We would ask the authors to provide sources and the neurological rationale for this framing as a binary classification task.**

**This is made even more problematic by the fact that data are not intrinsically labeled. The dichotomization is derived from an ad-hoc statistical procedure, namely, subjects are categorized based on the statistical significance of a t-test between the observed MEPs. Regarding the analysis of MEPs using peak-to-peak measures, it has to be noted that peak-to-peak measures are statistically non-robust and prone to the influence of noise and outliers.**

**We believe that analyzing TMS efficacy in their datasets as a regression problem could lead to better mechanistic insights as we find it reasonable to model graded responses to brain stimulation; if it is true that some people do not respond to treatment at all, zero-inflated models could prove to be a useful consideration.**

R1: We thank the reviewer for this insightful comment and for encouraging us to consider regression-based modeling. We agree that iTBS after-effects are, in principle, continuous and that continuous modeling can be valuable for mechanistic inference. However, we intentionally framed the primary analyses as a **classification** problem because (i) it matches the dominant **decision-oriented** formulation in the rTMS prediction literature, (ii) it yields results that are directly comparable to prior work (including the few neurophysiology-prediction studies), and (iii) it is more robust to the statistical properties of single-session neurophysiological change measures in typical TMS datasets.

From clinical and practical perspective, the vast majority of machine learning studies focusing on predicting rTMS effects, especially for clinical/behavioral endpoints, have categorized participants as responders vs non-responders (or remitters vs non-remitters) because the translational decision problem is often “who is likely to benefit?” rather than “how much will the benefit be?”. This binary distinction has become the dominant approach for clinical decision-making. This is explicitly reflected in multiple studies summarized in a recent review of the literature [1] confirming that the majority of ML studies in this domain utilize classification approaches to derive clinically actionable biomarkers. In particular, several studies operationalize response as a binary label derived from standard clinical thresholds (e.g., symptom reduction/remission criteria) and then evaluate AUC/accuracy/sensitivity/specificity rather than continuous-error metrics. This is not meant to imply a bimodal biology; rather, it reflects a decision-oriented endpoint used widely in the field (please refer to Table 1 in [1] for the full list of studies).

Most importantly, the few recent studies that have specifically attempted to predict neurophysiological outcomes (similar to our work) have also adopted classification-based frameworks. Both Katagiri et al. [2] and Cai et al. [3] utilized classification models to predict individual variability in iTBS after-effects. By adhering to this established methodology, we ensure that our findings regarding rsEEG complexity are directly comparable to these benchmarks.

We also acknowledge the reviewer’s point that regression could, in theory, extract graded information. In practice, however, single-session neurophysiological change scores (MEP- and TEP-derived) often exhibit substantial heterogeneity and heavy-tailed variability across individuals, including occasional extreme facilitation/suppression. Under these conditions, standard regression objectives (especially squared-error losses) can be disproportionately influenced by extreme values and can yield unstable out-of-sample performance unless the sample size is large and/or the target is strongly transformed and modeled with robust losses. Conceptually, converting the outcome to a classification target is an explicit bias–variance tradeoff: it sacrifices magnitude resolution but can yield a more stable learning target when the mechanistically meaningful question is whether detectable target engagement (facilitation vs suppression / change vs no detectable change) occurred. Relatedly, while it is not universally true that regression “always requires larger sample sizes,” in this specific setting (noisy outcomes, heavy-tailed changes, and low single-session reliability), learning a stable continuous mapping x→y typically requires more independent observations (or substantially stronger modeling constraints) than learning a thresholded/directional label. The core issue is effective signal-to-noise for *magnitude* prediction: when magnitude is weakly predictable and dominated by rare extremes, continuous estimators can become high-variance and generalize poorly even when directional/thresholded prediction remains feasible.

Finally, we would like to better explain our rationale for using statistical testing (pre- vs. post-MEP t-tests) to label participants. We agree that MEPs can be variable and require careful handling. In our dataset, however, the single-session MEP outcome is based on a large within-subject sampling distribution, not a small number of pulses. Each participant completed approximately 120–150 MEP trials per block, and thus ~360 trials per session across pre- and post-iTBS blocks (Methods; page 10, line 15–18). This high trial count substantially stabilizes estimation of the within-block amplitude distribution and reduces the influence of single-trial artifacts.

To further mitigate sensitivity to outliers, we removed extreme trials using a pre-specified criterion (excluding amplitudes exceeding 2.5 SD from the block distribution; Supplementary Methods; page 3, line 12–13). Importantly, the within-block variance is relatively small (distinct from the large *between-subject* variability in iTBS effects): across blocks, the mean within-block SD of peak-to-peak MEP amplitude was 1.20 μV, while the mean amplitude was 2.13 μV. A representative example of all trial waveforms (blue) and the block mean waveform (black) is shown in Figure 1, illustrating that the block-level MEP estimate is not driven by a small number of extreme trials.

Regarding the reviewer’s point that the data are “not intrinsically labeled” and that dichotomization is derived from an “ad-hoc” statistical procedure, we emphasize that our goal was not to claim a biologically bimodal distribution. Instead, the within-subject pre-vs-post test provides an objective, individual-specific criterion for whether a detectable physiological change occurred relative to that individual’s baseline under a fixed decision threshold (α = 0.05). Because the test is applied at the trial-distribution level within each session, it leverages the measurement structure of MEPs (many trials per block) and yields a reproducible operational definition of “detectable change” that is directly tied to the data-generating process. We agree that alternative continuous formulations can be informative; therefore, we additionally report a supplementary regression analysis for the key TEP outcome (below) to evaluate graded prediction without altering the main analysis plan.

Nevertheless, we agree with the reviewer that dichotomization has limitations. To address the reviewer’s suggestion that graded responses may be informative, we performed an additional regression-based analysis as a *supplementary* check, rather than re-analyses of the entire study. Specifically, we focused on one of our primary classification results (the best-performing TEP-based model in the Cross-Cohort Experiment) and evaluated whether the same predictor set could explain continuous variability in the corresponding TEP change metric. We used the feature subset identified during the Cross-Cohort TEP model-selection pipeline as the predictor set.

As shown in Figure 2 below, the histogram of the ratios of LMFP for the 100-131 ms window for the combined cohorts approximately follows the gamma, skewed to the right (x-axis represents the ratios, y-axis represents the frequency). Because the outcome is strictly >0 by construction (and shows no spike at 0), a zero-inflated model is not appropriate since zero-inflation is designed for targets with an *excess probability mass at exactly zero* (common in count data, or specific semi-continuous settings), whereas “non-response” in our data corresponds to ratios near ~1 (or small log-ratios), not to a pile-up at 0. For these reasons, we modeled the outcome using a Gamma GLM with a log link, which (i) respects the positive support of ratio outcomes and (ii) naturally accommodates right-skew and multiplicative effects. Next, we use the same training method used in the main text; we trained the GLM model on Cohort 1 and computed its regression performance on Cohort 2, as shown in Figure 3 (blue: mean-squared error, orange: mean absolute error and green: R^2^). The mean absolute error is around 0.33. Note that the R^2^ is actually negative, suggesting opposite signs between some predicted and actual ratios. Finally, to identify the neurophysiological response as facilitation or suppression, we classified the participants based on their ratio, with the participants classified as a responder if the ratio equals or exceeds 1, or else a non-responder. Then, we extracted the same classification performance metrics used in the main text and plotted it in Figure 4. The performance is slightly better than our main result for accuracy, sensitivity and f1 score (Figure 4d of the manuscript) but worse for specificity, area under the curve (AUC) of the receiver operating characteristic, AUC of the precision-recall curve and precision. Since the performance of GLM is heavily skewed with stronger sensitivity but weaker specificity, we prefer the balanced performance of the decision tree shown in the main manuscript (Figure 4d of the manuscript).

Figure 1. Plot of MEP trials from a representative subject in a pre-TBS Block. Blue lines represent individual trials and black line represents the averaged MEP.

Figure 2. Histogram of the ratios of TEP between post- and pre-iTBS protocol. The x-axis represents TEP ratios and the y-axis represents frequency (count in each bin).

Figure 3. Regression performance of GLM model with the response variable modelled as the gamma distribution and the log function as the link function. Blue bar represents mean squared error (MSE), orange bar represents mean absolute error (MAE) and green bar represents R^2^. Dark horizontal lines represent theoretical chance level based on sample class proportion. Thin vertical stripes within the bars represent confidence intervals computed by bootstrapping.

Figure 4. Classification performance based on classifying the predicted TEP ratios from the GLM model. Legends are the same as in Figure 4 of the main text.

**C2: We believe that the study might suffer from an inflated number of false negative non-responders as the authors use a two-sample t-test (instead of a paired t-test) for a repeated measures design. The too large variance estimate of the two-sample t-test will produce inflated p-values that might falsely label responders as non-responders. This is especially detrimental in connection with the classification problem from CS1 as responders and (falsely labeled) non-responders might share features that could predict treatment success but then would falsely lose predictive power.**

**Further, there is no specific reason why a p-value threshold of 0.05 should distinguish responders from non-responders. In fact, this threshold constitutes an additional, hidden parameter with possibly high leverage on the results and their interpretation, and is mere convention in frequentist statistics about the acceptable false-positive rate in the absence of any true effect. Even when considering the p-values as suitable dichotomization variable, the distribution of the t-statistics would be necessary to validate the assumption of a bimodal distribution.**

R2: We thank the reviewer for raising these statistical concerns. Please note that, although the overall design is repeated-measures at the subject/session level (pre vs post), a paired t-test is not defined at the trial level in our dataset because there is no meaningful one-to-one pairing between individual MEP trials in the pre- and post-iTBS blocks (trials are collected in separate blocks, with different counts across time points and cohorts). A paired t-test requires matched observations (e.g., each “pre” trial must correspond to a specific “post” trial), which would be arbitrary here and could introduce artificial structure into the variance estimate. Therefore, our responder labeling uses a two-tailed two-sample comparison of the pre- vs post-block trial-wise peak-to-peak amplitudes within each session, explicitly avoiding assumptions about directionality (facilitation vs suppression). Moreover, our labeling procedure is intentionally framed as a within-session detectability criterion (is there evidence that the post block differs from baseline for that individual?), rather than as a claim about discrete biological subtypes.

As such, the two-sample t-test is conducted between two blocks of MEP trials, approximately 120-150 trials per sample.

The use of 0.05 as the significance level is largely due to inertia as it provides an objective, individual-level criterion for detectable change in MEP responses. Keeping the significance level 0.05 allows our results to be compared with other results.

Please refer to our response R4 below where we addressed your comment about bimodal distribution.

**C3: In general dichotomization without a safety margin is problematic as it leads to a loss in discriminative power, especially when looking at graded effects. Without a safety margin around the decision threshold, insignificantly different subjects are classified as responders and non-responders, making it difficult to extract valuable predictors, as they are close in the feature space, muddying the waters effectively.**

**We would therefore like to see the distributions of MEP amplitudes and LMFP ratios that were used for either responder and non-responder population labelling or the labeling of facilitation or suppression.**

R3: We thank the reviewer for emphasizing an important limitation of dichotomization: observations close to the decision boundary can be assigned to different classes despite being practically indistinguishable, which can reduce discriminability and obscure predictors. Please note that the classifiers estimate the conditional distribution P(Y|X), not the marginal distribution P(X) or P(Y). As we explained in our previous response, this is not a concern for MEP analyses as we used statistical testing for class labelling. However, as we explained, this was not possible for TEPs. With that said, the distributions of the outputs largely follow gamma or log-normal distributions, with some exhibiting exponential distributions, a type of gamma distribution. Figure 5 shows the histogram of MEP ratios for the T5 block. Figure 6 shows the histogram of LMFP for a representative window.

To directly address the reviewer’s concern, we explicitly tested a **three-class formulation** that included a “no-change” band (ratio 0.9–1.1) around the boundary (conceptually aligned with the ~10% cut-off used in prior work). This three-class formulation significantly reduced the effective sample size per class and increased the complexity of the decision boundary relative to the available data. Consequently, none of the models achieved performance metrics above our pre-defined criteria (Accuracy/ROC AUC > 0.6 and sensitivity/specificity > 0.5).

Nevertheless, we agree this is a methodological limitation of the LMFP/TEP binary labeling, and we now explicitly acknowledg**e** in the Discussion that boundary-adjacent observations may reduce discriminative power and that future work should consider (i) larger samples enabling multi-class modeling, and/or (ii) continuous (regression) or probabilistic/ordinal approaches that better preserve graded effects.

Figure 5 Histogram of MEP ratios for the T5 block.

Figure 6. Histogram of LMFP ratios for a representative window.

**C4: When employing machine learning in neuroscience, interpretability of features matters at least as much as predictive accuracy. The authors claim that the study shows the superiority of complexity measures over regular time or frequency domain analyses. While complexity measures are an interesting upcoming analytical tool, we do not fully see that claim justified based on their data because the complexity measures are the only analysis features that make use of the full time series nature of the data.**

**We think the measures employed from time or frequency domain are insufficient. They, in contrast to the complexity measures, rely on heavy averaging and do not make sufficient use of the time series structure or trial structure of the data. Including both time series analyses (making use of the prototypical response beyond max amplitude point measures) and network features such as dynamic connectivity, phase amplitude couplings to characterize network state into the models might further increase interpretative, discriminative or predictive power for iTBS efficacy.**

**It remains vague how complexity measures based on scalp EEG in clinical applications of neurostimulation actually reflect endogenous brain activity, compared to how much is contributed by muscle or stimulation artifacts. Given these interpretative problems, the predictive power of complexity measures cannot support the scientific advancement of EEG-based TMS interventions.**

R4: We appreciate the reviewer’s suggestion to include network-level features such as dynamic connectivity and Phase-Amplitude Coupling (PAC). We agree that, in theory, these metrics offer a richer description of the brain's state. However, our decision to restrict the feature space to Spectral Power and Complexity was a deliberate methodological choice driven by robustness, interpretability, universality and simplicity of univariate features as opposed to the statistical constraints of high-dimensionality that come with multivariate features. We also clarify that our conclusions are not that “complexity is universally superior,” but rather that within the specific and widely used feature families we evaluated, rsEEG complexity features provided the strongest predictive signal for our outcomes under rigorous cross-validation.

We respectfully disagree that spectral power metrics are "insufficient" because they rely on averaging. Spectral powers computed from averaging allow us to capture the endogenous and steady-state brain oscillations, a widely used tool in the neuroscience community to study the brain activity. Unlike more complex metrics, spectral features map directly onto established physiological constructs found in decades of clinical and cognitive neuroscience. For example, delta activity is commonly associated with reduced vigilance and pathological slowing in neurological disease [4]; frontal-midline theta is repeatedly linked to cognitive control [5] and working memory demand [6]; alpha power is widely interpreted as reflecting thalamo-cortical rhythms [7], cortical “idling,” [8] and functional inhibition/attentional gating [9]; and sensorimotor beta is tightly connected to motor system state and movement suppression [10], and inhibitory–excitatory balance [11]. These band-limited measures therefore provide an interpretable and clinically meaningful characterization of intrinsic cortical state that is comparable across studies and cohorts.

To complement our steady-state features derived from the resting-state EEG, we included complexity measure, which was shown to capture both the endogenous and spontaneously evoked activity of the brain [12], a direct consequence of the time-structure and non-linear dynamics of resting-state EEG as you pointed out. In our results, complexity features (particularly coarse-grained multiscale distribution entropy) emerged as the most informative predictors among the candidate feature families we tested, suggesting that baseline temporal organization/nonlinearity of intrinsic activity contains information relevant to iTBS after-effects.

We agree dynamic connectivity / phase–amplitude coupling are promising directions. However, connectivity and PAC feature families introduce practical and scientific constraints that are non-trivial in our setting. First, scalp-level dynamic connectivity metrics often show lower and more variable test–retest reliability than band power, and there is substantial heterogeneity in how they are defined (choice of connectivity estimator, leakage correction, referencing, frequency definition, windowing, thresholding, graph construction), making them less standardized than spectral power measures. Second, they would dramatically expand the feature space in a way that increases overfitting risk and weakens interpretability without strong a priori constraints. With 63 channels, even a single static connectivity matrix yields 63×62/2 = 1,953 unique edges per frequency band; adding multiple bands, time windows (“dynamic” connectivity), or graph-theoretic summaries rapidly inflates dimensionality and introduces many correlated, partially redundant predictors. The same is true for PAC, which can be computed across multiple electrode pairs, phase–amplitude frequency pairings, and windowing choices. Third, there is no widely accepted mechanistic model specifying which networks or connections should change following single-session iTBS in healthy adults, making an unconstrained inclusion of large connectivity/PAC feature sets especially susceptible to “fishing” interpretations.

This concern is not merely conceptual: even with our current, deliberately constrained feature set, the model-selection step already required training **10,152 MEP models and 218,268 TEP models**. Adding high-dimensional connectivity/PAC families would substantially multiply the number of candidate models and comparisons. For these reasons, we prioritized a parsimonious and clinically grounded rsEEG feature set (power + complexity) while explicitly acknowledging connectivity/PAC as important future extensions.

We agree that complexity metrics must be interpreted cautiously in scalp EEG. However, in our study, complexity features were computed from resting-state EEG acquired prior to stimulation, so they cannot reflect stimulation artifacts. We also applied standard preprocessing to reduce non-neural contamination: noisy channels/segments were removed, ICA was used to identify and exclude ocular/muscle/cardiac components, and complexity estimates were computed on short, stationary segments (10 s) and averaged across segments to reduce sensitivity to transient noise.

In response to this comment, we have (i) clarified that our findings show complexity features were most informative within the evaluated feature families, not that they are inherently “superior” in general; (ii) expanded the rationale for emphasizing canonical band power features as the most established and interpretable rsEEG markers; and (iii) added dynamic connectivity/PAC feature families as explicit, hypothesis-driven future directions that will require larger samples and stronger a priori constraints to avoid an excessively high-dimensional feature space.

## Reviewer 3’s Comments:

Summary: The work is well motivated and the analyses presented feature a comprehensive list of tools applied. The need for external validation, i.e. an independent test set, is a sine non qua in traditional ML benchmarks; I am therefore happy to see this applied to clinical recordings, where data is much sparser. I commend the authors' rigorous approach to address the important issue of subject response variability, even more so if that means that results become non-significant. By doing so, this work highlights the importance of rigorous testing procedures, in order to accurately estimate the true clinical value of iTBS.

Overall, the paper is well written, but organization and presentation of the manuscript requires some work. I found several passages hard to comprehend, e.g. due to the large number of acronyms and unfamiliar terminology the authors use (see below).

With some revision, I will be able to recommend this work for publication.

**C1: The authors introduce two splits into train/test sets for their data: the cross-session split (experiment 1) and cross-cohort split (experiment 2). Why are separate analysis methods used for each of the two splits (statistical tests vs. training classifiers)? The authors motivate this with concept drift (page 20 line 21), but it did not become clear why the two dataset splits are paired to a specific analysis method.**

R1: We apologized for the confusion we’ve caused. We agree that our original presentation was confusing, and we have revised the manuscript to clarify the analytical workflow. Indeed, we did train classifiers in both Cross-Session Experiments and Cross-Cohort Experiments, with the results of the Cross-Session Experiment originally provided in Supplementary Methods and Materials to keep the manuscript concise. We now provide a brief description of the ML results in the main text added a clear statement in the results to direct readers to the Supplementary Methods and Materials for details of the Cross-Session results (page 20, line 1-9 of the revised manuscript).

The additional statistical tests and reliability analyses are not an alternative to machine learning, but rather diagnostic analyses intended to interpret the Cross-Session findings and to evaluate whether time-separated repeat sessions introduce instability in outcome labels and feature–outcome mappings.

By contrast, the Cross-Cohort split is designed to test external generalization across independent cohorts under near-identical protocols. Because “concept drift” is typically defined as a time-dependent change in p(y∣x) or the decision boundary, we reserve that term for the Cross-Session analysis and avoid using it to describe cross-cohort performance differences in the revised manuscript. Notably, the internal–external performance gap was smaller in the Cross-Cohort analysis than in the Cross-Session analysis, consistent with the idea that time-separated instability contributes to the larger drop observed in the Cross-Session setting. The remaining cross-cohort performance gap is instead discussed in terms of between-cohort generalization limits (and is addressed further in our response to the next comment).

**C2: Performance on the test set is generally reduced, for both the cross-session (p20l14) and cross-cohort (p22l21) analyses, and the authors suspect that is due to concept drift. In a strict definition, concept drift is a change in the decision boundary *occurring over time*. I understand that this may explain the performance drop in the cross-session split, but I am puzzled how concept drift could occur in the cross-cohort split. Even if we don't adhere to the strict definition above, but instead one where p(y|x) changes but p(x) does not (p13l5), it is not exactly clear whether it is p(y|x), or p(x) or p(y) which differ between train and test sets in the cross-cohort experiment. This argument requires further careful examination, as it appears to be one of main results underpinning the message of the paper. I'd like to see the reason of the performance drop identified; concept drift can be singled out through data augmentation or synthetic data, while the statistical tests of experiment 1 can be applied to the dataset split of experiment 2 to test for covariate and label shift.**

R2: We appreciate this important clarification. We agree with your point about the strict definition: concept drift refers to changes in the decision boundary (p(y∣x)) occurring over time. We have revised the manuscript to use “concept drift” only for the time-separated cross-session split (initial vs retest) and not for the cross-cohort split. Even though only Cross-Session Experiments’ splitting reflect the chronological nature, we applied statistical tests to both Cross-Session and Cross-Cohort Experiments to detect possible non-chronological shift in data distribution for Cross-Cohort Experiments. Please note that the gaps between training and test performance in Cross-Cohort Experiment are reduced relative to the Cross-Session Experiment, suggesting that concept drift explain the reduced performance gap but does not explain the remaining performance gap.

To diagnose the cause of performance drop in cross-cohort experiment, we’ve followed your suggestions and performed series of statistical tests for internal analysis. We’ve found that none of the spectral features have statistically different distributions between two cohorts, with only 7.7% of the complexity features have statistically different distributions between two cohorts. With respect to the MEP, none of the two blocks (T5 and T25) have statistically different class distributions, as assessed by the chi-squared test and with respect to the LMFP ratios, only 3 out of 43 different windows have statistically different distributions, as assessed by two-tailed Fisher exact tests. Collectively, these results rule out covariate shift and label shifts. Since the concept of change over time is non-applicable to Cross-Cohort Experiment, we have revised our interpretation of performance drop for cross-cohort experiment in the discussion

Since the test performance of the decision tree is lower than its training performance, this fits the classical symptom of overfitting. As mentioned in the original manuscript, the selected model is a decision tree that only use 3 out of the 9 provided features and the maximum depth is 4 nodes, suggesting a relatively simple non-linear model. For internal investigation of overfitting, we compared the performance of the exact same feature set, which consists of 9 features, with models of varying complexity, including a smaller decision tree with a maximum depth of 2 (as opposed to the maximum depth of 4 of our selected model). As shown in Figure 7, which is now in Supplementary Methods and Materials as Supplementary Figure S1, the internal validation performances for all models other than decision tree are poor, indicating that the models are limited-capacity in term of learning the training data. These models vary in level of complexity, from linear classifiers of logistic regression and linear discriminant analysis, to nonlinear classifiers of nearest shrunken centroids and Gaussian Naïve Bayes. Next, as shown in Figure 8, which is now in Supplementary Methods and Materials as Supplementary Figure S2, the performances of every models other than decision tree are roughly similar between internal validation and external validation, suggesting that these models are under-fitted. Lastly, for our internal post-hoc analysis, we’ve tried random forest, bagging ensemble learning, extra random forest, adaptive gradient ensemble learning, gradient boosting ensemble and extreme gradient boosting ensemble learning, with the decision trees as the weak learners in all ensemble methods, on the same feature set used in our decision tree. The results are shown in Figure 9. The performance profiles are all similar to our original decision tree, with the performance skewed to high sensitivity and low specificity. This suggested that the bottleneck is the data and not the model. We finally offer possible explanations in the revised manuscript, including insufficient sample size, invalid or unreliable measures of corticospinal and cortical excitability, invalid or unreliable experimental protocols for stimulating or recording MEPs or TEPs, unreliable rTMS protocols, etc on page 30 line 9-23, page 31 line 1-23 and page 32 line 1-15.

Finally, it’s not recommended to use data augmentation or synthetic data for test set to estimate generalization performance because it’s a form of data leakage.

Figure 7

Figure 8

Figure 9

**C3: This closely ties in with a question regarding the cross-cohort split: Splitting the train/test datasets by subjects instead of session makes sense, but (why) do the subsets need to consist of the two data collection cohorts (2018-2021, 2016-2019)? This may be a reason for the decrease in test performance; instead of concept drift, it may be an overall bias present within each data collection cohort. I would like to see a cross-subject analysis, with subjects sampled randomly from both data collection cohorts.**

R3: We thank you for this intuitive suggestion. Based on your suggestion to address study-wide bias in each data collection cohort, we’ve added a Cross-Subject Experiment to the manuscript where were randomly sampled 50% of the subjects (with both initial and retest sessions) from both cohorts for the internal validation set and all of the remaining subjects in both cohorts for the external validation set. Additionally, we’ve added 2 different variants of support vector machine just for this analysis. While the internal validation performance of the decision tree (assessed by cross-validation) is above 0.70 for all 7 metrics (Figure 10, now added to Supplementary Methods and Materials as Supplementary Figure S3), the external validation performance of the decision tree is unfortunately poor (Figure 11, also added to Supplementary Methods and Materials as Supplementary Figure S4), with some metrics below chance-level (short dark horizontal bars). In the revised manuscript, we offered two possible interpretations. First, this suggested that study-wide bias does not cause performance drop observed in the Cross-Cohort and Cross-Subject Experiments. However, another important possible explanation is that sampling subjects from both cohorts does not necessarily eliminate study-wide biases and thus it’s also possible that study-wide bias also still explain the performance drop observed in both Cross-Cohort and Cross-Subject Experiments. Importantly, either interpretations, we still conclude that the data is the bottleneck, not the model.

  

Figure 10

Figure 11

**C4: For experiment 2, the authors test a large range of classifiers (9) using cross-validation on the train set and testing on the test set. I commend this comprehensive analysis. I do wonder however how the different classifiers compare to each other? I could not find results for the ones which were not winners (LDA OA / decision tree), except for a mention in the supplement (see below).**

R4: We’ve answered this in R2, including interpretation and conclusions. Figure 7 shows the performance of internal validation (or training set) and Figure 8 shows the external validation performance (or test set).

**C5: In experiment 2, the best models chosen are chosen via highest ROC-AUC during cross-validation on the training set. This may not be the best metric to find a model which is able to generalize, as it only relies on data from cohort 1 for model selection. Do the winning models remain the same if models are selected by highest test performance or smallest drop in performance between training and testing?**

**R5:** We thank the reviewer for raising this important point about model-selection criteria. We agree that the choice of selection metric can influence which model is identified as “best” within the training cohort. However, selecting a model based on **external test-set performance** (or minimizing the train–test drop using the test set) is **not a valid model-selection strategy**, because it uses information from the held-out cohort to guide selection and would yield optimistically biased estimates of generalization.

In the main analysis, we therefore followed standard practice and selected models **using Cohort 1 only**, ranking candidates by mean ROC–AUC across repeated stratified cross-validation. To directly address the reviewer’s concern about metric dependence, we conducted post-hoc analysis to ensure that the model has both good sensitivity (true positives) and specificity (true negatives), we first filtered the models during internal validation with both sensitivity and specificity greater than 0.60 and then pick the final model based on ROC AUC. The model with the highest external validation ROC AUC is Logistic Regression with L2 Regularization trained on coarse-grained multiscale distribution entropy from all EEG channels based on the same 100-131 ms LMFP window used in the LMFP Cross-Cohort Experiment. The similarity in the feature set and labelling suggested that our model selection process is generally robust. Figure 12 shows the model (labelled as Lasso L2 CV) with the highest external validation ROC AUC performance but unfortunately and its internal validation performance (Figure 13) is weak across all metrics.

Figure 12

Figure 13

**C6: Furthermore, have the authors considered training a support vector machine? SVMs are less likely to overfit and may extract more underlying features from the data than LDA (given an appropriate kernel size). I would like to see results for an SVM. That may also clarify if overfitting or concept drift (see above) are at fault for decreased performance.**

R6: We’ve decided to add 2 different variants of support vector machine (linear and quadratic kernels) based on your C3 comment and found that SVMs didn’t improve the performance. Please refer to R3.

**C7: While the overall aim of the work is clear, the presentation of individual steps is at times confusing. In particular, nomenclature should be de-cluttered, and the presentation clarified:**

R7: We thank you for putting in the effort to understand our manuscript despite it being confusing and hard to follow. We’ve made several revisions across the entire manuscript and especially the Result Section to enhance readability, including streamlining transitions between paragraphs and sections and replacing a few acronyms with their full spellings (such as spTMS to single-pulse TMS, LM to left motor and RQS to regression quality score).

**C8: Several sentences are filled with acronyms, and introduce multiple concepts at the same time. Example: p13l10-13.**

R8: Similar to C7, we’ve polished our writing to make it more accessible.

**C9: In the definition of the paper, what is a "model"? In usual ML lingo, a model is the algorithm/classifier which is trained. But fig. 2 implies that it is the combination of feature group, categorization method and algorithm/classifier. As this definition only occurs in this caption, it was hard to understand the manuscript on my first readthrough. Please clarify across the manuscript.**

R9: Based on your suggestion, we’ve added a definition of a model early in the Method Section, on page 15 line 2-3.

**C10: On a similar note, on page 12, the authors define the training data for the classifier: features x and response variables y. It should be stated more clearly that x is the input and y is the label/target to the binary classifier; furthermore, "response variable" only appears this one time in the whole manuscript, and it is not clear that these are supposed to be the "responder/non-responder" classes. Please clarify in text. Including this in Figure 2 also would help clear up the confusion.**

R10: We’ve revised the manuscript, including updating Figure 2 for clarity. We’ve replaced the term response variable with the term label to avoid confusion.

**C11: p13l10: what are intraclass correlation coefficients?**

R11: We’ve added the definition and formula to the Method section in the revised manuscript.

**C12: The authors number their two experiments, 1 (cross-session) and 2 (cross-cohort), but these numbers are not referenced again at the appropriate points in the analysis (also, "cross-session" only reappears in the discussion, not results section).**

R12: We’ve revised the manuscript to remove multiple names for the same thing and only refer to the experiments as Cross-Session and Cross-Cohort instead of by number.

**C13: At several points, no explanation for experimental protocols is given. While the experimental parameters are likely well motivated by clinical experience, an outsider will benefit from briefly reporting the reasoning. Examples:
-- p10l1: "In both studies, single pulses TMS were administered at 120% of the RMT of the motor hotspot." How did you choose the value 120%?
-- p10l15: "In both studies, the iTBS protocol was applied to the motor hotspot at 80% of the AMT" How did you choose the value 80%?**

R13: We’ve added a reference to the safety guideline and recommendations provided by the International Federation of Clinical Neurophysiology, which provides the basis for the iTBS parameters.

**C14: - In ML lingo, "internal validation dataset" is known as "train set" (in this case, with cross-validation), and "external validation set" as "test set". It would be good to define this clearly in the manuscript.**

R14: For the Cross-Cohort Experiments, we used the term external validation, which while similar to test set, emphasize that the dataset used for testing is completely independent of the dataset used for training. Internal validation is also similar to training set but emphasized that it’s all done on a single study/dataset. For the Cross-Session Experiments, we used the term training and test sets instead. We’ve added definition to the method on page 15 line 15-18 of the revised manuscript

**C15: It took some time for me to understand that all classifications in this work are *binary*, using the labels "responder/non-responder" for MEP and "facilitation/suppression" for LMFP. It would be good to clear this up early, e.g. page 10 line 17. You may want to include a sketch of the training pipeline in fig. 2 (see below).**

R15: Based on your suggestion, we’ve emphasized that it’s a binary classification problem in the Abstract (page 2 line 15-16 of the revised manuscript) and the Background Sections (page 9 line 8-9 of the revised manuscript).

**C16: p21l15-15: "The performances are above chance level for all metrics. However, the confidence intervals for all metrics contain chance-levels." This statement is contradictory. The authors probably mean to say that the *mean* performance is above chance level. If so, please correct.**

R16: We’ve added the word “mean” to avoid confusion on page 24 line 1 of the revised manuscript.

**C17: p21l21-22: "To address possible concept drift, we change the way dataset is split in order to include both initial test and retest sessions during training" I found this sentence to be misleading, as concept drift is not *addressed* (i.e., solved), but rather *examined*. (there is also a typo, a "the" missing before "dataset")**

R17: We’ve incorporated both of your suggestions.

**C18: - Presentation of results for the cross-cohort split should be streamlined:**

**-- Tab. 1: The added value of this table, compared to Fig. 4, did not become apparent to me. Also, formatting of the table needs to be improved: confidence intervals should appear in the same line as the mean value. Instead of rows "a", "b" etc, denote which dataset (train/test) was used. For rows b and d, show differences in performance w.r.t. rows a, c.**

**-- It seems that the subsection "MEP Cross-Cohort Experiment" also repeats the numbers of Tab 1 and Fig 4. The same goes for the subsection "LMFP Ratios Cross-Cohort Experiment". If they do not add anything, I suggest removing the paragraphs citing data.**

**-- Move Fig 4 to be close to Tab 1.**

R18: Figures are now attached as separate files instead of together with the main text in the revised manuscript, as required by the journal. We want to present readers the options to see performance in both graphical and tabular forms. We are unable to put the confidence intervals in the same line as the mean values due to limited space.

**C19: - p16l5: It took me some time to understand what is meant by "sensitivity-(1-specificity) curve". Please typeset as an equation.**

R19: We’ve typeset the “sensitivity-(1-specificity) curve” as an equation but it still looks confusing. We were also confused when we first saw the term. Please see page 18 line 17 of the revised manuscript.

**C20: - p16l6: How is "recall" defined?**

R20: We’ve added clarification that recall is simply another word for sensitivity, however the standard term is precision-recall curve, not precision-sensitivity curve, unfortunately. Please see page 18 line 18 of the revised manuscript.

**C21: - p10l2: "spTMS", while understandable in context, is never defined.**

R21: We’ve removed abbreviation and spell out as single-pulse TMS.

**C22: Figures 2 and 4 are not placed near to where they are referenced.**

R22: Figures are now attached as separate files instead of together with the main text in the revised manuscript, as required by the journal. We’ve moved the figure captions closer to where they are referenced.

**C23: Figures and captions need to be improved. Fig. 1 is great, but most other figures do not make good use of space. They can be improved by removing whitespace, increasing fonts, and presenting a clearer flow of information.**

R23: We’ve made substantial improvement to Figure 2, 3 and 4 as well as their corresponding captions.

**C24: Please include vectorized graphics in all cases and homogenize font sizes of labels w.r.t. captions and main text.**

R24: Figures are now attached as separate files, in tif file format, instead of together with the main text in the revised manuscript, as required by the journal. The font size are homogenized now.

**C25: Fig. 2: The presentation could be improved. Why is the ROI selection part of "model generation"? (this relates to what the authors define as "model", see above) The figure would benefit from a 3-split presentation: Feature selection, label selection, classifier selection. Further, the workflow of the selection pipeline would benefit from an arrow from one step to the next (one table in top right connecting to the one in bottom left). Further, for the tables, different instances are stacked (top right, bottom center-right), but for the classifiers, they are separated vertically. Also, while all other choices are represented by an "x", the classifier selection is represented with 3 different arrows. Finally, you may want to picture how "features" and "labels" are fed into the classifier during training.**

R25: Your suggestion is extremely useful so we’ve made substantial improvement to Figure 2 by presenting it in 3 rows of panels.

**C26: Fig. 3 is very hard to parse, as it presents too much information, is too small, and pixelated.**

R26: We’ve also made substantial improvement to Figure 3 by removing MEP results and only presenting the LMFP results.

**C27: Fig. 4: The meaning of the terms "model selection" and "model validation" could be made clearer in this context. IIUC, the only difference is whether the internal validation dataset (cross-validation on train set) or external validation set (test set) are used. Please clarify.**

R27: We’ve changed the terms from model selection and model validation to internal validation and external validation to keep the terminology consistent. Please see updated Figure 4.

**C28: Fig. 4: Figure design can be improved but removing whitespace, increasing fonts. The classifiers (LDA OA/tree) should be prominently displayed instead of as x-tick marks.**

R28: We’ve incorporated all of your suggestions for the revised Figure 4.

**C29: Implications of the section "Results: MEP and LMFP Cross-Session Experiments" in the Supplement are not clear to me. What is the difference to the winning models (Table 1 in manuscript)? Please disambiguate.**

R29: We’ve clarified the difference in Cross-Session and Cross-Cohort Experiments as well as the order. The poor results of cross-session is the inspiration for conducting analysis for possible concept drift and Cross-Cohort Experiments are our updated experiments to handle possible concept drift.

References

1. Watts D, Pulice RF, Reilly J, Brunoni AR, Kapczinski F, Passos IC. Predicting treatment response using EEG in major depressive disorder: A machine-learning meta-analysis. Transl Psychiatry. 2022;12:332. https://doi.org/10.1038/s41398-022-02064-z

2. Katagiri N, Saho T, Shibukawa S, Tanabe S, Yamaguchi T. Predicting interindividual response to theta burst stimulation in the lower limb motor cortex using machine learning. Front Neurosci. 2024;18:1363860. https://doi.org/10.3389/fnins.2024.1363860

3. Cai G, Xu J, Ding Q, Lin T, Chen H, Wu M, et al. Electroencephalography oscillations can predict the cortical response following theta burst stimulation. Brain Res Bull. 2024;208:110902. https://doi.org/10.1016/j.brainresbull.2024.110902

4. Ip C-T, de Bardeci M, Kronenberg G, Pinborg LH, Seifritz E, Brunovsky M, et al. EEG-vigilance regulation is associated with and predicts ketamine response in major depressive disorder. Transl Psychiatry. 2024;14:64. https://doi.org/10.1038/s41398-024-02761-x

5. McFerren A, Riddle J, Walker C, Buse JB, Frohlich F. Causal role of frontal-midline theta in cognitive effort: a pilot study. J Neurophysiol. 2021;126:1221–33. https://doi.org/10.1152/jn.00068.2021

6. Hsieh L-T, Ranganath C. Frontal midline theta oscillations during working memory maintenance and episodic encoding and retrieval. Neuroimage. 2014;85 Pt 2:721–9. https://doi.org/10.1016/j.neuroimage.2013.08.003

7. Hindriks R, van Putten MJAM. Thalamo-cortical mechanisms underlying changes in amplitude and frequency of human alpha oscillations. Neuroimage. 2013;70:150–63. https://doi.org/10.1016/j.neuroimage.2012.12.018

8. Pfurtscheller G, Stancák A, Neuper C. Event-related synchronization (ERS) in the alpha band--an electrophysiological correlate of cortical idling: a review. Int J Psychophysiol. 1996;24:39–46. https://doi.org/10.1016/s0167-8760(96)00066-9

9. Morrow A, Elias M, Samaha J. Evaluating the Evidence for the Functional Inhibition Account of Alpha-band Oscillations during Preparatory Attention. J Cogn Neurosci. 2023;35:1195–211. https://doi.org/10.1162/jocn_a_02009

10. Barone J, Rossiter HE. Understanding the Role of Sensorimotor Beta Oscillations. Front Syst Neurosci. 2021;15:655886. https://doi.org/10.3389/fnsys.2021.655886

11. Illman M, Jaatela J, Vallinoja J, Nurmi T, Mäenpää H, Piitulainen H. Altered excitation-inhibition balance in the primary sensorimotor cortex to proprioceptive hand stimulation in cerebral palsy. Clin Neurophysiol. 2024;157:25–36. https://doi.org/10.1016/j.clinph.2023.10.016

12. Lau ZJ, Pham T, Chen SHA, Makowski D. Brain entropy, fractal dimensions and predictability: A review of complexity measures for EEG in healthy and neuropsychiatric populations. Eur J Neurosci. 2022;56:5047–69. https://doi.org/10.1111/ejn.15800
